# Editorial: Advances in Understanding and Rehabilitating Unilateral Spatial Neglect

**DOI:** 10.3390/brainsci13101437

**Published:** 2023-10-10

**Authors:** Arnaud Saj, Roberta Ronchi

**Affiliations:** 1 Department of Psychology, Montréal University, Montréal, QC H3C 3J7, Canada; 2 CRIR Institute Nazareth Louis-Braille, Longueuil, QC J4K 5G4, Canada; 3 Laboratory of Behavioural Neurology and Imagining of Cognition, Department of Fundamental Neurosciences, University of Geneva, 1202 Geneva, Switzerland; roberta.ronchi@unige.ch; 4 Neurology Service, Department of Clinical Neurosciences, Geneva University Hospital, 1211 Geneva, Switzerland

Unilateral Spatial Neglect (USN) is a frequent, very debilitating cognitive syndrome, in which patients fail to pay attention, perceive, and represent a part of the space in the side contralateral to the brain lesion [1]. While great progress has been made in defining the multifarious characteristics of this pathological condition and its brain correlates, our knowledge is still not exhaustive, and the understanding of spatial neglect continues to evolve. Still, researchers delve deeper into unravelling its complexities and improving effective rehabilitation strategies. 

The Special Issue “The Functional Neuroanatomy of Spatial Cognition and Neurorehabilitation in Neglect Syndrome” was conceived to update the current knowledge with a new perspective on the characteristics, brain correlates, and rehabilitation approaches for USN. The five publications presented here provide valuable insights into various facets of spatial neglect, shedding light on both its neural underpinnings and potential avenues for therapeutic intervention. 

Three studies explored and deepened the role of different brain circuits in spatial representation, using complementary techniques and populations, from fMRI to brain lesion approaches. 

Yann Cojan and colleagues [2] investigated the distinct spatial processing components contributing to hemi-neglect. Through lesion–symptom mapping and fMRI studies and combining information from the healthy and damaged brain, they highlighted the role of specific brain regions in various spatial tasks, such as bisection and visual search. Their findings emphasize the existence of separate attentional processes, each potentially affected to different extents in patients with neglect. 

Stephanie Leplaideur et al. [3] explored the neural bases of egocentric spatial representation in healthy participants using fMRI. Comparing two tasks testing the extracorporeal and corporeal spatial representation of body in space, they revealed the presence of parieto-occipital networks, with a right-hemispheric dominance, associated with both extracorporeal and corporeal tasks. Additionally, the corporeal component of spatial representation was linked to a more complex network, involving areas for motor imagery and body schema in the left hemisphere. These data suggest different levels of complexity in body–space representation. 

Finally, Nora Geiser and collaborators [4] discussed the role of the cerebellum in spatial processing. They presented a case study of visual neglect following a cerebellar stroke affecting the left PICA, which was detected only with more complex tasks and eye-tracking recording. This report suggests a potential modulatory role of the cerebellum in visual attention and raises questions about the sensitivity of neglect assessment tools, as well as of the timing of assessment post-stroke. 

As USN is a frequent disorder, which can bring about long-term negative effects on the functional outcome of patients [5], many studies have addressed the question about the most effective rehabilitation technique for it. Even if there is no consensus on the best approach, partially also due to the complexity of this syndrome and the involvement of many (compromised) mechanisms to rehabilitate, prism adaptation seems one of the more promising and frequently used approaches [6]. Two studies in this SI explore the neural correlates and methodology of prism adaptation for USN recovery. Olga Boukrina and Peii Chen [7] conducted a scoping review and meta-analysis of fMRI studies on prism adaptation in healthy individuals and patients. The review demonstrated distinct neural circuits engaged in prismatic adaptation between healthy and neglect-affected individuals, with neurologically unimpaired participants relying on cerebellar–parietal activations, and patients on the network based primarily in the left, unaffected hemisphere. The single-case study by Federica Albini et al. [8] also highlighted the role of specific neural damage in prismatic adaptation, as well as the impact of multisensory integration, analyzing the performance of a patient with bilateral atrophy of parieto-occipital cortices and apperceptive agnosia. Exploring the adaptation to prisms into different modalities, including a multisensory visuo-auditory condition, they found that the patient did not show the typical adaptation, as healthy participants did. However, when the after-effects subsequent to the adaptation were tested, the patient exhibited them despite lacking the adaptation phase, and the effect was comparable to the one of healthy participants when the adaptation was carried out in the multisensory condition. These data suggest a possible enhancement in the multisensory stimulation for increasing the rehabilitation effect of prisms. Moreover, their findings challenge the conventional understanding of the relationship between prism adaptation and after-effects, which subtend the mechanisms of recalibration and realignment, respectively, emphasizing the need for further investigation of the key characteristics of currently used rehabilitation methods.

Collectively, these studies contribute significantly to our knowledge of spatial neglect and its recovery. 

The reported results encourage the use of sensitive tasks for assessing different components of spatial representation and the definition of the specific damaged networks. Moreover, they highlight the intricate interplay of neural networks for processing distinct components of space and body exploration, including brain regions whose role is, up to now, controversial, such as the relationship between cerebellar lesions and neglect symptoms. Finally, they unveil some aspects of one rehabilitation technique currently used for USN rehabilitation, suggesting that spatial realignment can happen without the first recalibration explicit step of prismatic adaptation, possibly mediated by cerebellar structures. As we move forward, further research in this field promises to uncover more about the underlying mechanisms of spatial neglect. With each publication, we take another step towards a comprehensive understanding of this syndrome and its rehabilitation, offering hope for enhanced patient care and positive functional long-term outcomes, improving the lives of patients and their families.
